# Chronic endometritis multiplies the recurrence risk of endometrial polyps after transcervical resection of endometrial polyps: a prospective study

**DOI:** 10.1186/s12905-024-03221-w

**Published:** 2024-06-26

**Authors:** Jing Huang, Xiao You, Zijun Zhao, Xiaorui Jiang, Dacheng Qu

**Affiliations:** 1 Department of Obstetrics and Gynecology, Affiliated Hospital of North Sichuan Medical College, No 63, Wenhua Road, Nanchong, 637000 China; 2 Non-invasive and Micro-invasive Laboratory of Gynecology, Affiliated Hospital of North Sichuan Medical College, Nanchong, 637000 China

**Keywords:** Recurrence, Chronic endometritis, Endometrial polyps, Transcervical resection of endometrial polyps, CD138 expression by endometrial polyps

## Abstract

**Background:**

To investigate the impact of chronic endometritis (CE) on the recurrence of endometrial polyps (EPs) in premenopausal women after transcervical resection of endometrial polyps (TCRP).

**Methods:**

This prospective study enrolled 507 women who underwent TCRP between January 1, 2022 and December 31, 2022. The patients were divided into a CE group (*n* = 133) and non-CE group (*n* = 374) based on the expression of CD138 in the endometrium. The EP recurrence rate at 1 year after TCRP was compared between the CE and non-CE groups and between groups with mild CE and severe CE. The impact of CD138 expression by resected EPs on EP recurrence also was investigated.

**Results:**

The EP recurrence rate at 1 year post-TCRP was higher in the CE group than in the non-CE group (25.6% vs. 10.4%) and also higher in the severe CE group than in the mild CE group (34.5% vs. 18.7%). Additionally, the EP recurrence rate was higher among patients with CD138-expressing EPs than among those with EPs lacking CD138 expression (30.5% vs. 6.5%). The odds ratio (OR) for EP recurrence in the CE cohort compared with the non-CE cohort was 3.10 (95% confidence interval [CI] 1.84–5.23) after adjustment for EP number and precautions against EP recurrence. The ORs for EP recurrence in patients with mild CE and severe CE were 2.21 (95%CI 1.11–4.40) and 4.32 (95%CI 2.26–8.26), respectively. Similarly, the OR for EP recurrence in cases with CD138-expressing EPs relative to cases with EPs lacking CD138 expression was 6.22 (95%CI 3.59–10.80) after adjustment for EP number and precautions against EP recurrence.

**Conclusions:**

CE multiplied the recurrence rate of EPs in premenopausal women after TCRP, and this effect positively correlated with CE severity. CD138 expression by EPs also was associated with a higher risk for EP recurrence.

## Background

Endometrial polyps (EPs) are benign overgrowths of the endometrium that protrude into the uterine cavity in the forms of single or multiple tipped or untipped growths that contain varying numbers of glands, mesenchyme, and epithelium-covered blood vessels [1]. EPs represent one of the most common pathologies in gynecology, occurring from reproductive age to post-menopause, with a prevalence ranging from 7.8 to 41% in different populations [1, 2]. The pathogenesis of EPs has not been fully elucidated but has been mainly linked to hormone dependence, inflammatory stimulation, and family history [3]. The most common clinical symptoms of EPs are abnormal uterine bleeding (AUB) [4], recurrent pregnancy loss [5], and recurrent implantation failure [6].

Chronic endometritis (CE) is an inflammatory disease characterized by persistent localized endometrial infection with infiltration of plasma cells in the endometrial stroma [7]. Immunohistochemical (IHC) staining to detect CD138 cell-specific surface antigens is used to diagnose CE with good accuracy and sensitivity [8]. The clinical manifestations of CE are essentially the same as those of EPs, consisting of AUB, infertility, and poor pregnancy outcomes, including recurrent pregnancy loss and repeated implantation failures [9]. Intrauterine pathogen infections are thought to be the main cause of CE [10]. CE leads to changes in the microenvironment of the uterine cavity, mainly in the intrauterine microbiota spectrum [8] and in intrauterine immunity [11]. These changes can lead to the formation of EPs. Peng et al. [12] found that the occurrences of both solitary polyps and multiple polyps are positively associated with CE.

Transcervical resection of endometrial polyps (TCRP) is the gold standard treatment method for EPs [13], but postoperative EP recurrence remains a major problem. Reported recurrence rates vary greatly, ranging from 1.39 to 45.5% at 1 year after TCRP [14–16]. At present, the mechanism of EP recurrence has not yet been clarified, although research has identified the number of EPs and previous history of TCRP as definite independent risk factors for the recurrence of EPs after TCRP [14]. Unfortunately, these factors are essentially unchangeable, and thus, do not offer intervention opportunities to reduce the risk of EP recurrence. One study found that the recurrence of EPs may be related to inflammation and immunity [17]. Also, in a retrospective study, Qu et al. [18] showed that CE is an independent risk factor for EP recurrence in premenopausal women after TCRP. In their study, the recurrence rate of EPs was significantly higher in patients with CE than patients without CE at 1 year after TCRP. In contrast to the above-mentioned unchangeable risk factors for EP recurrence, CE is relatively simple to treat, with cure rates exceeding 70% with a course of antibiotics [19]. Therefore, further research is needed to explore the impact of CE on the recurrence of EPs after TCRP.

In this prospective study, we investigated whether CE increases the risk of EP recurrence in premenopausal women after TCRP and further investigated the impacts of CE severity and the expression of CD138 on EPs on the recurrence of EPs at 1 year post-TCRP.

## Methods

### Study design

Between January 1, 2022 and December 31, 2022, premenopausal women with EPs who were admitted to the Affiliated Hospital of North Sichuan Medical College to undergo TCRP were prospectively enrolled. This study was approved by the ethics committee of the Affiliated Hospital of North Sichuan Medical College (No. 2022ER383-1). Patient data were anonymized and protected according to national standards. The patients were tested for endometrial CD138 expression and EP CD138 expression after TCRP. The patients were divided into the CE group (endometrial CD138 positive) and non-CE group (endometrial CD138 negative) based on endometrial CD138 expression status, and both groups were followed up prospectively. The recurrence rates of EPs at 1 year after TCRP were compared between the CE group and non-CE group. The recurrence rates of EPs at 1 year after TCRP also were compared between subgroups with differing CE severity (based on CD138 staining intensity) and between subgroups with and without CD138 expression on resected EPs.

The inclusion criteria were: pathologically confirmed diagnosis of EPs after TCRP, premenopausal, and sufficient endometrial specimen for immunohistochemical analysis of CD138 expression to identify CE. The exclusion criteria included: previous history of TCRP, endometriosis, hyperplasia, or endometrial cancer diagnosed pathologically after TCRP, antibiotic treatment for 14 days after TCRP, planning childbirth within 1 year, unexpected pregnancy during follow-up, and loss to follow-up.

### TCRP and immunohistochemistry

TCRP was performed in the follicular phase with a bipolar plasmakinetic resection system under intravenous anesthesia using a 3-mm 12° inside rigid hysteroscope and an 8.5-mm outside sheath (Olympus, Tokyo, Japan). EPs were completely removed at their base using the plasma electrode ring. All operations were performed by senior physicians (ZY Xia and DC Qu). EP specimens and endometrial specimens were collected from each patient during TCRP, fixed in 10% neutral formalin solution, dehydrated in alcohol, and serially sectioned at 4-µm thickness after routine paraffin embedding. The tissue sections were baked in a 60–65℃ over for 2 h, then were soaked in environmental protection transparent agents I and II for 10 min each, anhydrous ethanol solutions I and II for 5 min each, 95% ethanol, 90% ethanol, 85% ethanol, 80% ethanol for 3 min each, and rinsed slowly with water for 5 min. After antigenic thermal repair and endogenous peroxidase blocking, sections were subjected to primary antibody incubation, re-warming, secondary antibody incubation, addition of streptavidin–biotin complex drops, color development, and Mayer’s hematoxylin staining and re-staining. Then neutral gum was used to seal the slices. Microscopic photographs were taken and preserved, and the results were recorded.

**Fig. 1:**
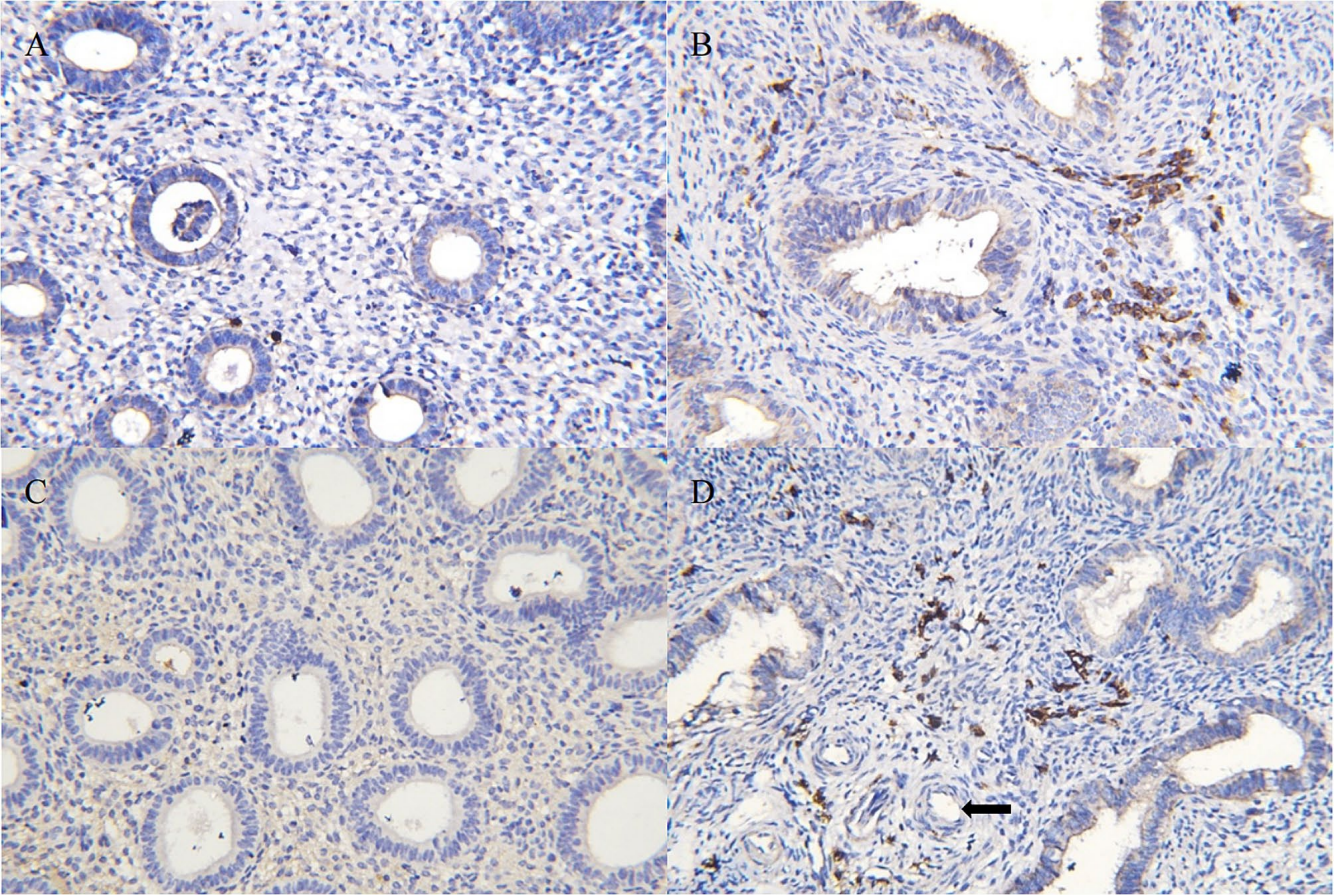
CD138 expression on endometrium and EPs. (**A**) Mild CE (1–4 CD138-positive cells per 10 high power fields). (**B**) Severe CE (≥ 5 CD138-positive cells per 10 high power fields). (**C**) Lack of CD138 expression on endometrium. (**D**) CD138 expression in endometrium and EP sections. A thick-walled blood vessel was visible (arrow). EP: endometrial polyp; CE: chronic endometritis

CE was diagnosed by at least one CD138-positive cell per 10 high-power fields in this study, as widely used in other studies. At least 50 high-power fields were examined for each specimen [18, 19]. CE cases were categorized as mild CE (1–4 CD138-positive cells per 10 high power fields) and severe CE (≥ 5 CD138-positive cells per 10 high power fields [20] (Fig. 1). The criteria used for EP expression of CD138 was consistent that same as that used for CE (Fig. 1).

### Follow-up

Hysteroscopy was performed 1 year after TCRP to detect recurrence of EPs without any anesthesia using a 3-mm 30° inside rigid hysteroscope and a 4.5-mm outside sheath (Olympus, Tokyo, Japan). Normal saline solution at 100 mm Hg pressure was used to distend the uterine cavity. The hysteroscopic body entered the uterine cavity sequentially along the vaginal orifice, cervical external ostium and internal ostium without contact, cervical traction, or cervical dilation. The opening of the right fallopian tube, the opening of the left fallopian tube, the fundus, and the walls of the uterine cavity were checked in sequence, and the cervical canal was rechecked as the hysteroscope was removed.

### Statistical analyses

For all statistical analyses, we used SPSS version 26.0 (SPSS, Inc, Chicago, IL, USA). Measurement data conforming to continuous normal distribution are expressed as mean ± standard deviation, and those not conforming to a normal distribution are expressed as median (P25, P75). Count data are expressed as frequency or rate (%). Between-group comparisons of data conforming to a normal distribution were performed using the two-sample independent t-test, and those of data not conforming to a normal distribution were performed using the nonparametric Mann-Whitney U-test. Count data were compared between two groups using the chi-square test, and EP recurrence rates were compared between two groups using the Pearson chi-square test. Correlation between EP expression of CD138 and endometrial expression of CD138 was tested by Spearman correlation analysis. Univariate and multivariate logistic regression analyses were used to identify risk factors for the recurrence of EPs, and variables with *P* < 0.1 on univariate logistic regression analysis were included in the multivariate logistic regression analysis. Odds ratios (ORs) and 95% confidence intervals (CIs) were calculated for the association of each variable with the recurrence of EPs, with *P* < 0.05 indicating statistical significance.

## Results

### Comparison of general and clinical features of patients who underwent TCRP

**Fig. 2:**
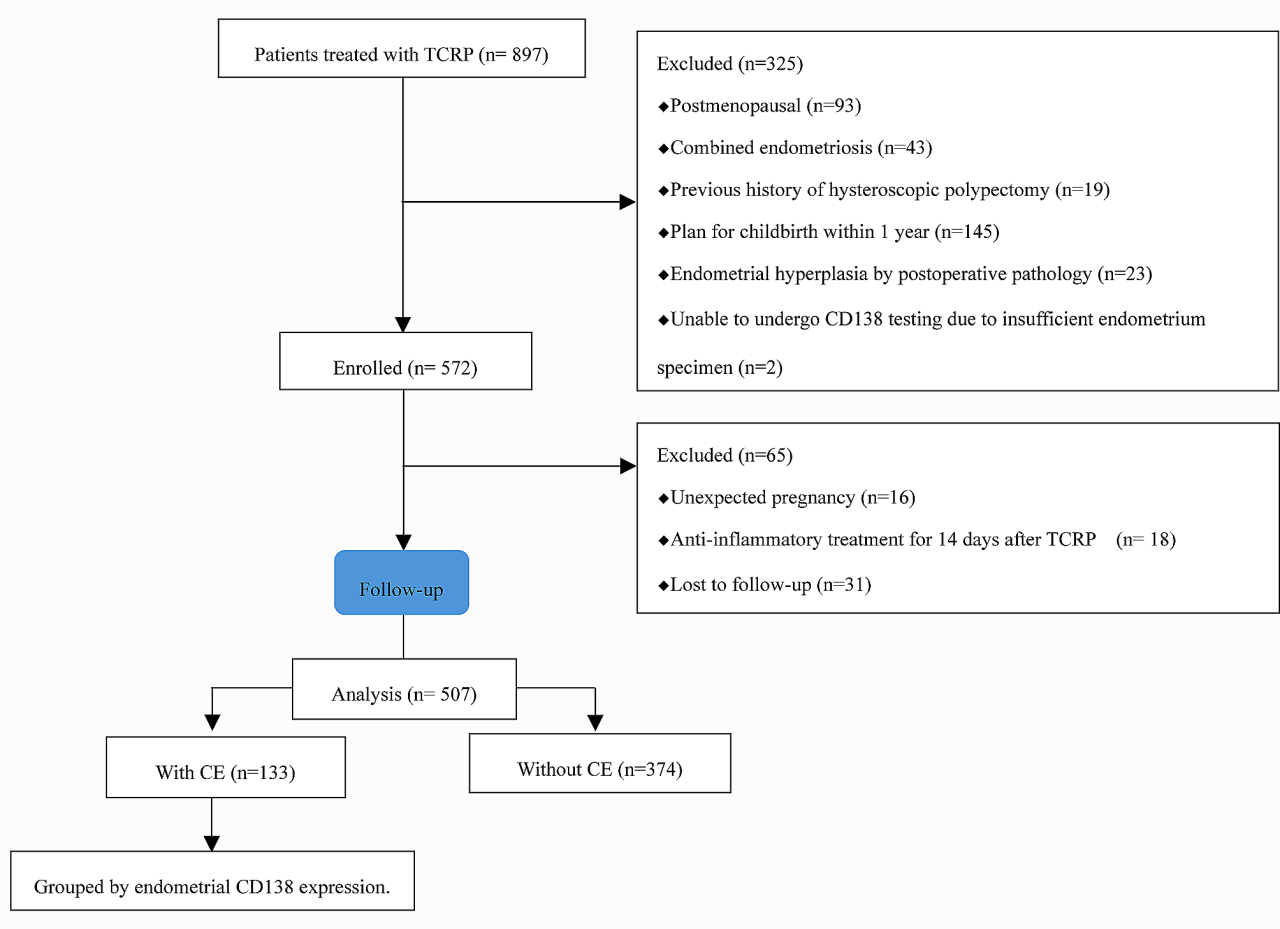
Flow chart of patient inclusion in the study

During the 1-year study period, a total of 897 women who underwent hysteroscopic polypectomy were enrolled in this study. Of them, 572 cases met the inclusion criteria, while 325 cases were excluded. Another 65 cases were excluded during the 1 year follow-up after TCRP, including 16 cases with unexpected pregnancy, 18 cases received anti-inflammatory treatment for more than 14 days after TCRP, and 31 cases lost to follow-up. Finally, 507 cases were included in the final statistical analysis, with 133 cases assigned to the CE group and 374 cases to the non-CE group (Fig. 2). The prevalence of CE in the study population was 26.2% (133/507). The general and clinical features of the two groups are presented in Table 1. The incidence of AUB in the CE group was significantly higher than that in the non-CE group (*P* < 0.05). No significant differences were observed in the other general and clinical features of the two groups (*P* > 0.05).

**Table 1:** Baseline characteristics of the enrolled EP patients with and without CE.

Characteristics	EPs with CE (*n* = 133)	EPs without CE (*n* = 374)	*P*
Age, y (median (Q3-Q1))	33 (7)	33 (7)	0.534
BMI (kg/m^2^), n (%)			0.715
< 28.0	9 (6.8%)	22 (5.9%)	
≥ 28.0	124 (93.2%)	352 (94.1%)	
EP number, n (%)			0.870
Solitary	36 (27.1%)	104 (27.8%)	
Multiple	97 (72.9%)	270 (72.2%)	
EP size (cm), n (%)			0.322
< 1	21 (15.8%)	48 (12.8%)	
≥ 1, <2	107 (80.4%)	300 (80.2%)	
≥ 2	5 (3.8%)	26 (7.0%)	
EP position, n (%)			0.118
Anterior	23 (17.3%)	50 (13.4%)	
Posterior	28 (21.0%)	110 (29.4%)	
Side	10 (7.5%)	13 (3.5%)	
Cornual	11 (8.3%)	26 (6.9%)	
Multiple	61 (45.9%)	175 (46.8%)	
ER expression, n (%)			0.121
Positive	122 (91.7%)	324 (86.6%)	
Negative	11 (8.3%)	50 (13.4%)	
PR expression, n (%)			0.897
Positive	113 (85.0%)	316 (84.5%)	
Negative	20 (15.0%)	58 (15.5%)	
AUB, n (%)			0.001
Present	98 (73.7%)	215 (57.5%)	
Absent	35 (26.3%)	159 (42.5%)	
Precautions against EP recurrence, n (%)			0.470
Yes	41 (30.8%)	103 (27.5%)	
No	92 (69.2%)	271 (72.5%)	

Abbreviations: EP: endometrial polyp; CE: chronic endometritis; BMI: body mass index; ER: estrogen receptor; PR: progesterone receptor; AUB: abnormal uterine bleeding

### Influence of CE and severity of CE on recurrence of EPs after TCRP

**Fig. 3:**
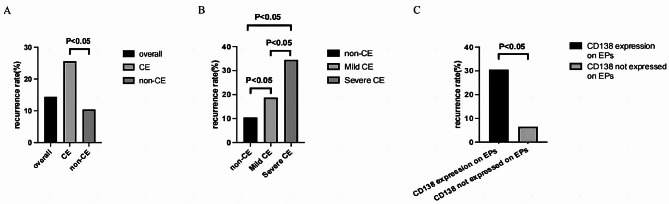
Recurrence rates of EPs at 1 year after TCRP in patient groups. (**A**) Comparison of EP recurrence rates between the patient groups with and without CE. (**B**) Comparison of EP recurrence rates in subgroups with mild and severe CE. (**C**) Comparison of EP recurrence rates in subgroups with and without CD138 expression by EPs. EP: endometrial polyp; CE: chronic endometritis

At the 1-year monitoring stage after TCRP, a total of 73 women had experienced EP recurrence, including 34 cases in the CE group and 39 cases in the non-CE group. The corresponding recurrence rates of EPs in the CE group and non-CE group were 25.6% and 10.4%, respectively, with an overall recurrence rate of 14.4% (Fig. 3). The EP recurrence rate was significantly higher in patients with CE than in those without CE (*P* < 0.001). Further analysis showed that the risk of EP recurrence at 1 year after TCRP was almost 2-fold greater in patients with CE than in those without CE (OR = 2.95, *P* < 0.001; Table 2).

**Table 2:** Univariate and multivariate logistic regression analyses of factors association with EP recurrence in patients with CE.

Characteristics	OR (95%CI)	*P*
Univariate analysis		
Age	1.02 (0.98–1.06)	0.383
Obesity	0.87 (0.30–2.58)	0.807
EPs size	1.13 (0.64-2.00)	0.671
EPs position	1.04 (0.86–1.27)	0.671
ER	1.13 (0.51–2.49)	0.761
PR	1.03 (0.52–2.06)	0.936
AUB	0.76 (0.46–1.26)	0.291
Precautions against EP recurrence	0.50 (0.27–0.94)	0.033
Multiple EPs vs. solitary EP	3.07 (1.49–6.36)	0.002
CE	2.95 (1.77–4.92)	0.000
Multivariate analysis		
CE	3.10 (1.84–5.23)	0.000
Multiple EPs vs. solitary EP	3.13 (1.50–6.55)	0.002
Precautions against EP recurrence	0.47 (0.25–0.91)	0.024

Abbreviations: EP: endometrial polyp; CE: chronic endometritis; ER: estrogen receptor; PR: progesterone receptor; AUB: abnormal uterine bleeding

On the subgroup analysis based on CE severity, EP recurrence occurred in 14 cases in the mild CE group and 20 cases in the severe CE group. The corresponding recurrence rates of EPs with mild CE and severe CE were 18.7% and 34.5%, respectively. The recurrence rate of EPs was higher in patients with severe CE than in patients with mild CE (*P* = 0.038; Fig. 3). Notably, the EP recurrence rates in patients with mild CE or severe CE were both higher than that in patients without CE.

Univariate and multivariate logistic regression models were used to identify factors associated with the recurrence of EPs after TCRP in premenopausal women. Univariate analysis showed that the recurrence of EPs was not affected by age, obesity, EP size, EP position, estrogen receptor (ER) expression, or progesterone receptor (PR) expression. However, significantly higher EP recurrence rates were observed in women with multiple EPs (*P* < 0.05) and women with CE (*P* < 0.05), and a lower EP recurrence rate was observed in women with a history of precautions against EP recurrence (*P* < 0.05), compared with the rates in patients without the respective conditions (Table 2). Multivariate analysis confirmed that the risk of 1-year EP recurrence after TCRP was 2-fold greater when CE was present than when not (OR = 3.10, 95% CI 1.84–5.23, *P* < 0.001; Table 2). On subgroup analysis according to CE severity, the risk of 1-year EP recurrence after TCRP was 1-fold greater with mild CE (OR = 2.21, 95% CI 1.11–4.40, *P* = 0.023) and 3-fold greater with severe CE (OR = 4.32, 95% CI 2.26–8.26, *P* < 0.001) than that in patients without CE (Table 3).

**Table 3:** Multivariate logistic regression analysis of the association of CE severity with EP recurrence

Characteristics	OR (95%CI)	*P*
Mild CE	2.21 (1.11–4.40)	0.023
Severe CE	4.32 (2.26–8.26)	0.000
Multiple EP vs. solitary EP	3.09 (1.47–6.47)	0.003
Precautions against EP recurrence	0.50 (0.26–0.97)	0.040

Abbreviations: EP: endometrial polyp; CE: chronic endometritis

### Influence of CD138 expression by EPs on EP recurrence after TCRP

Subgroup analysis based on CD138 expression by EPs showed that CD138 expression by EPs correlated with CE (CD138 expression on endometrium) with a Spearman correlation coefficient of 0.307 (*P* < 0.001). Overall, 167 cases had CD138-expression EPs, and of these, 51 cases experienced EP recurrence within 1 year after TCRP, for an EP recurrence rate of 30.5%. Comparatively, of 340 cases with EPs lacking CD138 expression, 22 cases experienced EP recurrence, for an EP recurrence rate of 6.5%. Accordingly, the recurrence rate of EPs was higher in patients with CD138-expression EPs (*P* = 0.038; Fig. 3). Multivariate analysis showed that the risk of EP recurrence within 1 year after TCRP was 5-fold greater for cases with CD138 expression detected in EPs (OR = 6.22, 95% CI 3.59–10.80, *P* < 0.001; Table 4).

**Table 4:** Univariate and multivariate logistic regression analyses of the association of CD138 expression by EPs and EP recurrence

Characteristics	OR (95%CI)	*P*
Univariate analysis		
CD138 expression by EPs	6.36 (3.69–10.94)	0.000
Multivariate analysis		
CD138 expression by EPs	6.22 (3.59–10.80)	0.000
Multiple EPs vs. solitary EP	2.92 (1.38–6.22)	0.005
Precautions against EP recurrence	0.50 (0.26–0.98)	0.043

Abbreviation: EP: endometrial polyp

## Discussion

### Main findings of the study

This prospective study demonstrated that the recurrence rate of EPs at 1 year after TCRP was significantly higher in premenopausal women with CE than in those without CE. Additionally, severe CE was associated with a greater risk of EP recurrence than mild CE. These findings indicate that CE is a risk factor for the recurrence of EPs in premenopausal women after TCRP. The OR for EP recurrence in the CE group compared with non-CE group was 3.10, after adjustment for EP number and precautions against EP recurrence. Moreover, the OR for EP recurrence with the detection of CD138 by EPs was 6.22, after adjustment for EP number and precautions against EP recurrence. These findings indicate that CD138 expression by EPs is also a risk factor for EP recurrence after TCRP.

### Implications and comparison with literature

Local inflammation of the endometrium is contributing factor to the formation and recurrence of EPs, while CE involves chronic persistent inflammation of the endometrium that often coexists with EPs [9, 12, 19, 21]. Our previous retrospective study showed that CE may be a risk factor for EP recurrence in premenopausal women who underwent TCRP [18]. In this prospective study, to better evaluate the impact of CE on EP recurrence, patients with definite external confounding risk factors for EP recurrence, including previous history of TCRP and endometriosis, were excluded. The recurrence rate of EPs in the current study population was 14.4% at 1 year after TCRP, which is consistent with the rates reported in previous studies [15, 18, 22]. However, the EP recurrence rates differed between patients with CE and those without, reaching 25.6% in patients with CE and only 10.4% in patients without CE. These rates are nearly consistent with those observed in our previous retrospective study [18]. Together, these findings confirm that CE is a harmful factor for the recurrence of EPs. Imbalance in the microenvironment of the uterine cavity may be an important factor in the occurrence of intrauterine diseases, such as EPs and CE. First, the intrauterine microbiota spectra in cases with CE or EPs show significant changes in the relative abundance distribution of species, with predominantly non-lactobacilli observed in both CE and EP cases. *Staphylococcus*, *Gardnerella*, *Atopobium*, *Streptococcus*, *Peptostreptococcus*, *Chlamydia*, *Fusobacterium*, *Acinetobacter*, and others were previously found to be related to CE and EPs [23]. Secondly, the alteration of intrauterine immunity caused by CE may be another factor contributing to the recurrence of EPs [24–28].

From the subgroup analysis based on the severity of CE, the EP recurrence rates were 18.7% in patients with mild CE and 34.5% with in patients severe CE. These EP recurrence rates in both mild CE and severe CE cases were significantly higher than that in patients without CE. Additionally, the EP recurrence rate was significantly higher in patients with severe CE than in those with mild CE. Severe CE involves more inflammation in the basal layer of the endometrium and can persist for a longer period, making it more likely to cause recurrence of EPs.

Univariate logistic analysis was performed to assess the relationship between EP recurrence and multiple potential influencing factors, including age, obesity, EP size, EP position, EP number, ER expression status, PR expression status, history of precautions against EP recurrence, CE, and CD138 expression by EPs. On the univariate logistic analysis, multiple EPs, precautions against EP recurrence, CE, and CD138 expression by EPs were associated with the potential recurrence of EPs (*P* < 0.05). The OR for EP recurrence in the CE cohort compared with the non-CE cohort was 2.95 (95% CI 1.77–4.92). After adjustment for EP number and precautions against EP recurrence, this OR was 3.10 (95% CI 1.84–5.23). This value is almost consistent with the HR of 3.06 calculated in our retrospective study [18]. Similarly, after subgroup analysis based on CE severity, the ORs for EP recurrence were 2.21 (95%CI 1.11–4.40) with mild CE and 4.32 (95% CI 2.26–8.26) with severe CE.

The correlation analysis showed that CD138 expression by EPs was correlated with CE. Comparatively, the EP recurrence rates were 30.5% in patients with CD138-expressing EPs and 6.5% in patients with EPs that lacked CD138 expression. Similarly, the OR for EP recurrence with expression of CD138 by EPs compared with no CD138 expression by EPs was 6.36 (95% CI 3.69–10.94). After adjustment for EP number and precautions against EP recurrence, the OR was 6.22 (95% CI 3.59–10.80). A possible explanation for this effect of CD138 expression by EPs is that EP formation in such cases may be caused by inflammation. Therefore, patients who have persistent chronic inflammation may be more likely to experience EP recurrence after TCRP.

### Strengths and limitations of the study

These results of this study indicate that CE is an independent risk factor for the recurrence of EPs after TCRP. To the best of our knowledge, this is the first study to report the effect of CE severity and the effect of CD138 expression by EPs on the recurrence of EPs after TCRP. The EP recurrence rate was significantly higher in patients with severe CE than in those with mild CE. Additionally, subgroup analysis based on the presence of CD138 expression by EPs showed that the EP recurrence rate was significantly higher in patients with CD138-expressing EPs than in those with EPs lacking CD138 expression. In contrast with unchangeable factors, such as EP number and previous TCRP history, CE can be cured by treatment. Thus, to better prevent recurrence of EPs after TCRP, routine testing for CE and for CD138 expression by EPs during surgery is recommended. If positive, antibiotic treatment should be given after TCRP. However, owing to the lack of consensus regarding antibiotic treatment plans, the cure rate for CE with one course of antibiotic treatment fluctuates greatly, ranging from 28.0 to 92.8% [29]. We recommend two commonly used regimens, doxycycline alone or levofloxacin combined with tinidazole for 14 days, because the cure rates achieved with one course of these treatments were 72.6% [18] and 89.8% [29] in randomized controlled trials, respectively. However, it is currently unclear whether CE treatment will reduce the recurrence rate of EPs and achieve a lower recurrence rate of EPs consistent with that in non-CE patients. Further prospective studies are needed to verify this.

The present study has some limitations. First, this was a single-center study, and the proficiency of TCRP surgery may affect the recurrence of EPs. Meanwhile, the incidence of EPs and postoperative recurrence rates may vary among different regions. The role of CE in the recurrence of EPs needs to be further confirmed by multi-center studies with large sample sizes, and the potential mechanism responsible for the harmful effects of CE remains to be investigated. Secondly, only the time point of 1 year after TCRP was monitored. Monitoring at multiple time points may more accurately describe the recurrence trend of EPs, although it would increase the time and economic burden on patients. Finally, this study was an observational study, without consideration of the interventional effects of antibiotic treatment for CE. Whether the impact of CE on the recurrence of EPs can be eliminated by treatment with antibiotics should be further explored.

## Conclusions

In conclusion, this study demonstrated that CE is an independent risk factor for the recurrence of EPs in premenopausal women after TCRP. Severe CE was associated with a higher risk for EP recurrence than mild CE, and CD138 expression by EPs also was associated with a higher risk for EP recurrence. More prospective studies are needed to further explore the effect of CE on the recurrence of EPs after TCRP.

## Data Availability

The datasets are available from the corresponding author on reasonable request.

## Abbreviations

EP: Endometrial polyp
CE: Chronic endometritis
TCRP: Transcervical resection of endometrial polyps
